# *Granulicatella adiacens* infective endocarditis in pregnancy: diagnostic contribution of metagenomic sequencing—a case report

**DOI:** 10.1128/asmcr.00237-25

**Published:** 2026-02-24

**Authors:** Aude Vasselin, Victor Scavazzin, Jean-Philippe Talarmin, Claudie Lamoureux, Marion Pérès, Hervé Le Bars, Marie-Sarah Fangous, Clémence Beauruelle, Séverine Ansart, Geneviève Héry-Arnaud

**Affiliations:** 1 Department of Infectious Agents, CHU Brest 26990, Brest, France; 2 Univ Brest, Inserm, EFS, UMR 1078, GGB 27002 , Brest, France; 3 Department of Infectious Diseases, Centre Hospitalier de Cornouaille 55151, Quimper, France; 4 Department of Infectious Diseases, CHU Brest 26990, Brest, France; 5 Laboratory of Microbiology, Centre Hospitalier de Cornouaille 55151, Quimper, France; 6 Center for Biome Analysis and Microbiota (CBAM), CHU Brest 26990, Brest, France; Rush University Medical Center, Chicago, Illinois, USA

**Keywords:** infective endocarditis, pregnancy, *Granulicatella adiacens*, cell-free DNA, metagenomic next-generation sequencing, blood culture-negative endocarditis

## Abstract

**Background:**

*Granulicatella adiacens* is a fastidious Gram-positive coccus and is a rare but recognized cause of infective endocarditis. Infective endocarditis during pregnancy is uncommon but carries substantial maternal and fetal risk. Plasma metagenomic analysis of microbial cell-free DNA has emerged as a complementary diagnostic tool in culture-negative infections.

**Case Summary:**

We describe a 35-year-old pregnant woman with known mitral valve prolapse who presented at 21 weeks of gestation with an acute ischemic stroke. Initial etiological work-up, including transesophageal echocardiography, was unremarkable. Ten days later, she re-presented with left-arm pain and neurologic symptoms. Repeat echocardiography revealed multiple mitral vegetations compatible with infective endocarditis. Despite multiple sets of prolonged-incubation blood cultures and extensive serological testing, all microbiological investigations remained negative. Empirical intravenous ceftriaxone was initiated based on the working diagnosis of HACEK endocarditis. A plasma metagenomic cell-free DNA test ultimately identified *G. adiacens*, which was suspected to have entered the body through dental treatment received a few weeks earlier. Ceftriaxone was continued given the favorable clinical response, with vegetation resolution, troponin decline, and uncomplicated term delivery of a healthy infant.

**Conclusion:**

This case illustrates the diagnostic challenges of culture-negative infective endocarditis in pregnancy and underscores the value of plasma microbial cell-free DNA sequencing as a complementary tool when conventional methods fail. It also emphasizes the need to repeat echocardiography when clinical suspicion remains high and raises the question of antibiotic prophylaxis for high-risk dental procedures in pregnant women with underlying valvular heart disease.

## INTRODUCTION

*Granulicatella adiacens* is a facultatively anaerobic, Gram-positive coccus belonging to the nutritionally variant streptococci (with *Abiotrophia defectiva*). The genus *Granulicatella* currently includes three validly published species (*G. adiacens*, *G. elegans*, and *G. balaenopterae*). *Granulicatella adiacens* is a low-abundance constituent of the oral microbiota, especially dental plaque, but it can also be detected in the nasopharyngeal, gastrointestinal, and genital tract microbiota (1, 2). Although uncommon, it is a recognized cause of bacteremia and infective endocarditis. Its recovery in blood cultures is often difficult because of fastidious growth requirements and poor growth on standard media (3).

Infective endocarditis remains challenging to diagnose due to frequently non-specific clinical manifestations, imperfect imaging sensitivity, and, in approximately 10% of cases, microbiological failure despite blood cultures (4, 5).

Endocarditis during pregnancy is exceptionally rare, below 0.01% of pregnancies, but carries substantial maternal and fetal morbidity, further complicating diagnosis (6–10).

When conventional investigations are inconclusive, metagenomic next-generation sequencing (mNGS) of microbial cell-free DNA (cfDNA) in plasma has emerged as a promising complementary tool for etiologic diagnosis in culture-negative endocarditis (11–15).

We report a case of *G. adiacens* infective endocarditis in a pregnant woman in whom etiologic diagnosis was obtained only through plasma microbial cfDNA sequencing.

## CASE PRESENTATION

**Fig 1:**
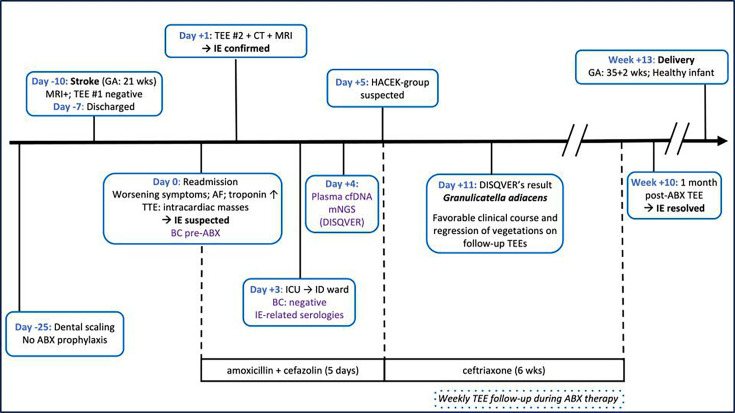
Timeline of clinical course and diagnostic strategy in culture-negative infective endocarditis. ABX, antibiotics; AF, atrial fibrillation; BC: blood cultures; cfDNA, cell-free DNA; CT, computed tomography; GA, gestational age; HACEK, *Haemophilus*, *Aggregatibacter*, *Cardiobacterium*, *Eikenella*, *Kingella*; ICU, intensive care unit; ID, infectious diseases; IE, infective endocarditis; mNGS, metagenomics; MRI, magnetic resonance imaging; TEE, transesophageal echocardiography; TTE, transthoracic echocardiography.

A 35-year-old woman with mitral valve prolapse with mild mitral regurgitation presented to the emergency department at 21 weeks of gestation with acute right-sided weakness (Fig. 1). Cerebral magnetic resonance imaging (MRI) showed a left-sided ischemic stroke. Intravenous thrombolysis was not performed because of the ongoing pregnancy. Antiplatelet therapy was initiated, and the patient was admitted to the neurovascular unit. The etiological workup, including transesophageal echocardiography (TEE), was unremarkable and did not reveal any embolic source. The patient recovered normal neurological function and was discharged home 3 days after admission.

Ten days after the stroke, she returned to the emergency department complaining of left-arm pain, headache, and vomiting. She was tachycardic, hypotensive, but afebrile. Electrocardiography showed atrial fibrillation. Laboratory tests revealed marked troponin elevation and leukocytosis with neutrophils at 9.7 G/L (standard between 2.5 and 7 G/L). A transthoracic echocardiography (TTE) showed a large thrombus attached to the mitral valve, highly suggestive of infective endocarditis. Retrospective review of the initial echocardiography revealed subtle early lesions. On admission day, she developed sudden right hemiparesis. A repeat MRI showed three new ischemic lesions in the left sylvian area. Because an intracardiac thrombus could not be confidently distinguished from infective endocarditis, therapeutic anticoagulation was started together with empiric intravenous antibiotics (amoxicillin 12 g/day and cefazolin 6 g/day), and she was transferred to the intensive care unit (ICU). She did not require vasopressor support. A new TEE was performed the following day, showing four mobile masses attached to the myocardial surface (largest 10 mm) and a tissue-like mass arising from the left ventricular wall without valvular involvement. The other valves were normal; no abscesses or thrombi were observed; and ventricular functions were good. The chest computed tomography scan and cardiac MRI confirmed these findings. Given the clinical picture combining ischemic stroke and the rapid onset of vegetations, the final diagnosis was infective endocarditis. The elevated troponin levels were attributed to a poor tolerance of atrial fibrillation.

Two to three sets of blood cultures (BD BACTEC Plus aerobic and anaerobic bottles; 8–10 mL per bottle) were obtained before antimicrobial initiation and incubated for 15 days at 37°C (endocarditis protocol) in a BD BACTEC FX 200 System; all remained negative for bacterial growth. After a multidisciplinary discussion, it was decided to treat the patient with antibiotics alone without surgery, as there was no evidence of cardiac, hemodynamic, or septic intolerance. Empiric intravenous dual antibiotic therapy was, therefore, continued.

The patient remained clinically stable, and obstetric ultrasound remained normal. After 48 h in the ICU, she was transferred to the infectious disease department. An extended microbiological assessment was performed, including multiple sets of prolonged-incubation blood cultures and serology for *Brucella* spp., *Coxiella burnetii*, and *Bartonella* spp.

At this stage, despite multiple sets of blood cultures (including those obtained prior to antimicrobial initiation) and an extended serologic work-up, no microbiological documentation was available. In this context, the multidisciplinary team considered that the bacteria responsible belonged to the HACEK group or a related group and, therefore, switched antibiotic therapy to intravenous ceftriaxone 2g/day, as recommended by the European Society of Cardiology (ESC) (16), after 5 days of treatment by amoxicillin and cefazolin.

In the absence of documentation, and because valve surgery was not indicated, the valve tissue was not accessible for culture or broad-range PCR; hence, the multidisciplinary team decided to request plasma microbial cfDNA mNGS DISQVER (Noscendo GmbH, Reutlingen, Germany) to accelerate etiologic diagnosis and support antibiotic de-escalation during pregnancy. DISQVER is a CE-IVD marked algorithm that uses unbiased metagenomic shotgun sequencing data from cfDNA isolated from patient plasma samples. More than 16,000 species of DNA microorganisms are included in the database, of which around 1,500 are classified as pathogens. The assay provides organism identification but does not provide phenotypic susceptibility testing or antimicrobial resistance profiling. The test was ordered on hospital day 4, and the report was returned 7 days later, positive for *G. adiacens* identified alone. The final diagnosis was *G. adiacens* infective endocarditis in a patient with pre-existing valvular heart disease, most likely secondary to dental scaling performed a few weeks earlier. Given the favorable clinical course and reassuring findings on follow-up cardiac ultrasonography after 7 days of ceftriaxone therapy (following an initial 5-day course of amoxicillin and cefazolin), ceftriaxone was pursued for a total duration of 6 weeks.

Follow-up TEEs were performed on a weekly basis for 4 weeks, showing a reduction in vegetation size. Pregnancy follow-up remained normal.

The patient gave birth vaginally after induction at 35 weeks and 2 days; delivery was uncomplicated, and the newborn was healthy.

The last TEE was performed 1 month after the end of antibiotic treatment, showing the complete disappearance of the vegetation.

## DISCUSSION

Infective endocarditis during pregnancy is rare but associated with substantial maternal and fetal morbidity and mortality, with reported maternal mortality of 11–33% and fetal mortality of 14–29% (6–10). *Granulicatella adiacens* is a very rare cause of pregnancy-associated infective endocarditis (7, 8, 10).

The patient had a pre-existing mitral valve prolapse categorized as an intermediate-risk lesion in the 2023 ESC guidelines and, thus, did not qualify for antibiotic prophylaxis before dental procedures (16). Nevertheless, she underwent dental scaling a few weeks before symptom onset, a plausible entry point for *G. adiacens* (1, 2, 17–25). Pregnancy-related hemodynamic and immunologic changes may further predispose to invasive infection (6–10, 16, 26). Whether pregnancy should influence decisions about antibiotic prophylaxis for high-risk dental procedures in women with structural heart disease remains unknown.

Nutritionally variant bacterial species, including *G. adiacens* and *A. defectiva*, have been associated with aggressive endocarditis characterized by large vegetations, frequent embolic events, and a high rate of early valve surgery (27). In a large cohort of bloodstream infections caused by streptococcal species and related *Streptococcus*-like organisms, *G. adiacens* was classified as a high-risk species for concomitant infective endocarditis, with estimated endocarditis rates of at least 10% and up to nearly 50% when three or more blood culture bottles were positive (28). These data support a low threshold for echocardiographic evaluation in patients with *G. adiacens* bacteremia. In addition, because these organisms are not *Streptococcus* from a genetic standpoint, some molecular blood culture identification panels relying on pan-*Streptococcus* targets may not detect them; this should be considered when endocarditis is suspected, and routine molecular panels are negative.

In our patient, the initial TEE at the time of the first stroke was interpreted as normal, and vegetations were clearly visualized only 10 days later, although retrospective review revealed subtle early lesions. Experimental models indicate that vegetation formation occurs rapidly once bacteremia is established (29, 30), suggesting that the delay in echocardiographic diagnosis more likely reflects limited sensitivity of imaging than delayed vegetation formation. The ESC guidelines recommend repeating echocardiography within 5–7 days when clinical suspicion of infective endocarditis remains high despite an initially negative examination (16, 31), a principle well illustrated by this case.

Blood cultures remain the cornerstone of etiologic diagnosis in infective endocarditis, but 5–10% of cases are blood culture-negative because of prior antibiotics or fastidious organisms (4, 5, 30, 31). Although modern automated blood culture systems are often able to detect *Granulicatella* spp., subsequent recovery on solid media and downstream testing may be challenging because these organisms may require enriched conditions (e.g., chocolate agar or blood agar supplemented with pyridoxal/vitamin B6 or L-cysteine). Therefore, when *Granulicatella* infection is suspected, the use of supplemented media and close interaction between clinicians and the microbiology laboratory can be critical to maximize recovery and enable susceptibility testing. Traditional adjunctive methods also include serology for *C. burnetii*, *Bartonella* spp., *Brucella* spp., and other fastidious pathogens and broad-range PCR on the resected valve tissue (4, 5, 30, 31). This last approach may be impractical when surgery is not indicated or is contraindicated.

Plasma microbial cfDNA mNGS offers a noninvasive alternative for pathogen detection in culture-negative endocarditis (11–15). Several studies have shown that such assays can identify plausible pathogens in a substantial proportion of patients with suspected infection and that results lead to changes in antimicrobial management in 10–30% of cases, particularly in deep-seated infections or after prior antibiotic exposure (13, 14). In our patient, the DISQVER plasma test detected *G. adiacens* and enabled the endocarditis documentation, which had failed with the usual methods, as well as the identification of the dental entry point. From an analytical standpoint, plasma cfDNA mNGS performance depends on pathogen burden and timing relative to antimicrobial exposure. Because it is a sequencing-based detection of DNA fragments, results can be influenced by contamination signals and may occasionally detect organisms not responsible for the clinical syndrome; conversely, false negatives may occur when DNA burden is low. Therefore, cfDNA mNGS is best viewed as complementary to conventional investigations and should be interpreted alongside Duke criteria, imaging, and the overall plausibility of the detected organism. Finally, the lack of phenotypic susceptibility data remains a key limitation; antimicrobial optimization must rely on guideline-based regimens, known susceptibility patterns, and careful clinical/echocardiographic monitoring.

Pregnancy imposes additional constraints on antimicrobial choice. Combination regimens, including a beta-lactam (penicillin/ampicillin or ceftriaxone) plus gentamicin, are frequently recommended for *Granulicatella* endocarditis, with vancomycin as an accepted alternative regimen (16, 27). In our patient, prolonged aminoglycoside therapy was avoided because of concerns regarding fetal toxicity (32). In the absence of initial microbiological documentation, the infection was first managed as possible HACEK endocarditis, and ceftriaxone was selected as step-down therapy after initial amoxicillin–cefazolin, given its favorable pregnancy safety profile (32, 33) and with intensive echocardiographic follow-up. As plasma cfDNA mNGS subsequently identified *G. adiacens* 7 days after the switch to ceftriaxone, the patient had already improved clinically, and follow-up TEE showed clear regression of vegetations, supporting continuation of ceftriaxone monotherapy. *G. adiacens* displays variable beta-lactam MICs, including occasional reduced susceptibility to ceftriaxone (34–37). In the absence of susceptibility testing (an inherent limitation when diagnosis relies on cfDNA mNGS alone), this approach requires close clinical and echocardiographic monitoring, and this case report should not be generalized as evidence supporting monotherapy in all cases.

In summary, this case emphasizes three key messages: (i) *G. adiacens* should be recognized as a high-risk cause of infective endocarditis, particularly in patients with dental manipulation and underlying valvular disease; (ii) in pregnant women with structural heart disease who present with ischemic stroke or other embolic events, infective endocarditis must be considered, and echocardiography should be repeated when initial imaging is negative, but suspicion remains high; and (iii) plasma microbial cfDNA mNGS can be decisive in establishing the etiologic diagnosis of blood culture-negative infective endocarditis and guiding safe, targeted therapy when surgery is undesirable or contraindicated.
